# Working as simulated patient has effects on real patient life – Preliminary insights from a qualitative study

**DOI:** 10.3205/zma001041

**Published:** 2016-05-17

**Authors:** Anne Simmenroth-Nayda, Gabriella Marx, Thorsten Lorkowski, Wolfgang Himmel

**Affiliations:** 1 University Medical Center Göttingen, Department of General Practice/Family Medicine, Göttingen, Germany; 2 University Medical Center Göttingen, Postdoctoral Fellow, Clinic for Palliative Medicine, Göttingen, Germany

**Keywords:** patient simulation, clinical competence, undergraduate medical education, physician-patient relations, patient participation

## Abstract

**Background: **Persons who simulate patients during medical education understand the routines and the underlying script of medical consultations better. We aimed to explore how simulated patients (SPs) integrated this new understanding into their daily life, how this work affected their private life as patients, and what we can learn from these changes for concepts of empowerment.

**Design, setting, and participants:** A qualitative interview study. All SPs of Göttingen medical school who had been working longer than three semesters (n=14) were invited and agreed to take part in an open interview about their daily experience with real doctors. Documentary method was used to identify the main issues. Several cases were chosen according to maximum contrast and analysed by in-depth analysis to provide vivid examples of how simulations may affect the real life of the SPs as patients.

**Results: **Our analysis revealed three main changes in the behaviour of SPs as real patients. They were more attentive, had a better understanding of the circumstances under which doctors work, and acted more self-confidently. From the selected cases it became apparent that working as a SP may lead to a constant and significant decrease of fear of hospitals and medical procedures or, in other cases, may enable the SPs to develop new abilities for giving feedback, questioning procedures, and explanations for real doctors.

**Conclusion: **working as a simulated patient seems to be well-suited to understand own progression of diseases, to increase self-responsibility and to a confident attitude as patient.

## Zusammenfassung

**Hintergrund: **Simulationspatienten (SP), die über Jahre am Unterricht mit Medizinstudierenden beteiligt sind, haben - im Vergleich zu realen Patienten – einen anderen Blick auf die ärztliche Konsultation, insbesondere auf kommunikative Aspekte. Daher war es Ziel dieser Studie zu untersuchen, wie SP diese Erfahrungen in ihr tägliches Leben integrieren und ob bzw. wie ihre realen Arzt-Patienten-Kontakte von den Erfahrungen im Unterricht beeinflusst werden.

**Methoden: **Es handelt sich um eine qualitative leitfadengestützte Interviewstudie. Alle 14 SP, die seit mindestens drei Semestern in unseren studentischen Kursen tätig waren, wurden zur Studie eingeladen. Die Interviews wurden in zwei Schritten analysiert: (1) Herausarbeitung von relevanten Themen durch Anwendung der formulierenden Interpretation der Dokumentarischen Methode nach Bohnsack, (2) eine Typenbildung auf Basis von Fallbeschreibungen, die gleichzeitig Handlungsmuster und -strategien illustrieren sollte.

**Ergebnisse:** Alle SPs nahmen an der Befragung teil. Die Analyse zeigte eine Veränderung im Arztkontakt vor allem bei drei zentralen Themen: eine größere Aufmerksamkeit in der Konsultation, mehr Verständnis für die ärztliche Arbeitssituation sowie gesteigertes subjektiv wahrgenommenes Selbstbewusstsein bei der Formulierung eigener Bedürfnisse. Die Fallgeschichten zeigen, dass die SP-Tätigkeit Ängste reduzieren kann, Defizite in Konsultationen transparent und kommunizierbar macht und SPs ermöglicht, gezielte Rückmeldungen an Ärzte zu geben.

**Zusammenfassung: **Die Tätigkeit als SP dürfte – im Sinne des „patient empowerment“ – zu einem besseren Kenntnisstand des eigenen Krankheitsverlaufs, einer größeren Verantwortungsübernahme, einer stärkeren Kontrolle des Krankheitsgeschehens und zu einem sichereren Auftreten als Patient führen.

## 1. Background

Working with simulated patients (SPs) is a widely accepted method for effective teaching and assessment in medical schools [1], [2], [3]. With the help of SPs, students learn manual skills as well as communication skills in a protected environment, including feedback from a patient perspective. SPs as a tool in medical education are welcome by both students and teachers; the effectiveness in teaching has been proven [4]. 

In addition to many positive effects on medical education, the persons who simulate patients understand the routines and the underlying script of medical examinations better. Previous studies suggest that experiences as SP, especially the activity in role-playing and feedback-training, influence the role of the SPs as real patients. Some previous studies have investigated the scope of this influence on a person’s behaviour as a patient [5], [6], [7], [8], [9]. Apart from some negative effects such as exhaustion, nervousness, or being displeased with their own efforts, the results showed that SPs develop a more differentiated view on their doctor-patient encounter or that they observe the communication skills of their own general practitioners (GPs) in more detail than other patients do. Moreover, SPs see themselves as more active when communicating with a health-care professional, which speeds up their recovery [10], [11]. Because of the gain in knowledge in medical topics, SPs are also more apprehensive about their own symptoms and disorders [12]. 

### 1.1. Aims of the study

 In addition to previous studies, which predominantly used surveys or focus groups [1], [6], [7], [9], [13], [14] to record changes in the life of an SP, we aimed at exploring in more detail and depth how SPs integrated their new experiences into their daily life, how this work affected their private life as patients, and what these changes teach us about concepts of empowerment.

## 2. Methods

### 2.1. Context and Sampling

Working with simulated patients as a teaching method was introduced at Göttingen medical school in 2005. Since then, all SPs have been trained and supervised by one of the authors (AS). Similar to most other medical schools, the main components of our SP training are communication skills such as history taking, breaking bad news, or counselling and risk-communication as well as assessment in objective structured clinical examination (OSCEs) and the medical school admission-procedure [1], [4], [15]. 

To ensure that the study participants had professional skills, we only invited the 14 SPs who had been working with third- and fourth-year students longer than three semesters and in addition had attended OSCEs at least four times per semester. They all agreed to take part in our study. All participants had to give written informed consent; it was possible to withdraw this at any time. The ethics committee of the University of Göttingen approved the study protocol (no 12/4/08).

#### 2.2. Data collection

To stimulate narratives of how the SPs dealt with their work, we developed an interview guide using open ended questions focusing on three main areas:

1. poor and good elements in our lessons,
2. teaching of the doctor-patient relationship in our course, and
3. the SPs’ experience with their real doctors.

In this paper we focus on the third area. All interviews were conducted by the same person (TL). 

After a short introduction of the study aim, we created a relaxed and familiar atmosphere to give the participants the opportunity to narrate their experiences. If the participants did not address the third question of their own volition in the course of the interview, they were asked: “What happens when you are a patient in real life? Please tell me about your daily experiences”.

After piloting the interview changed slightly. TL conducted all the interviews, they were audio-taped and transcribed verbatim; participants were pseudonymised.

#### 2.3. Data analysis

The semi-structured interviews should consider the main points of the interview-partners, so the documentary method according to Bohnsack [16] ,[17] seemed to be the best approach. The advantage of this method is the possibility to asses both – without methodological limitations – the broadness of topics and also the implicit ideas which are underlying the spoken words. 

The interviews were analysed in two ways:

1. To analyse central topics mentioned in the interviews, we used the first step of the documentary method according to Bohnsack, his concept of “formulating interpretation”. After reading each transcript, all narrations were summarized, and main topics as well as sub-topics were formulated using an inductive approach. This step refers to the content of the interview and provides a broad overview of what was said in the interview.
2. On the basis of the first step of analysis, we then identified several cases that represented quite distinct ways of “handling the work as a SP according to maximum contrast. These passages were analysed with the second step of Bohnsacks approach, the “reflecting interpretation”. We concentrated on “how something is said”, who was involved in the events, when and where the events took place and which feelings were reported in order to illustrate both different types of SPs and different types of transferring new skills into real-life consultations.

## 3. Results

### 3.1. Participants

All eligible SPs (n=14) agreed to take part in the study, nearly all of them were female (12/14). They were between 24 and 70 years old and had been working as SP for between two and six years. The interviews lasted between 20 and 55 minutes and took place in our department or at the home of the SPs.

#### 3.2. Experiences of SPs as patients in real life: main topics 

In the interviews, the SPs freely talked about their daily experiences as a patient in real life. The many simulated consultations they took part in while they were SPs seemed to have activated a learning process that led to (a higher) awareness of structure, rules, and content of consultations in daily life. Three main topics emerged in our analysis of the SPs’ practical knowledge in real consultations with doctors: they were more attentive, had a better understanding of the circumstances under which doctors work, and acted more self-confidently. Although these topics are presented separately here, in daily practice they are closely related. 

##### Being more attentive

Training in simulation and the skills as SP obviously helped our interview partners to better understand the structure of the doctor-patient communication. Within the consultation, SPs no longer only fill the role as a patient seeking for help, but also act as persons who are familiar with attentive evaluation. This attentiveness enabled the SPs to have a critical view of details of the consultation and the GPs’ behaviour, which led them to adopt a critical attitude.

SP 2: “Well, what is really clear to me now, somehow, so just my GP [general practitioner] (...) when you are consulting him, he is always asking a lot of round-about questions somehow, how is your situation, and so on, and in the past I thought: oh my God, I am ill, that‘s got nothing to do with it. But now: (...) through these role-plays I have realized, ok: the social history and all these things… (...) that‘s why he is asking, and those are the ideas he is thinking of.”

SP 9: “Yes, of course, I check the way how he (GP) enters the room. That is the result of these – these role-plays. How is he entering the room? Does he shake my hand? Does he look at me when shaking hands? Or is he doing something else meanwhile? Does he allow me to say something?” 

SP 4: ”It does happen, on occasion, that I view my doctor with different eyes, as it were, and think: now here he goes again, just almost falls into the examination room, didn’t even greet me properly, or lounges in his chair, or doesn’t even look at me or continuously interrupts what I’m saying.“

##### Better understanding of doctors

Through working with medical staff as a part of the medical training and curriculum, SPs had a more detailed idea of what it means to work as a doctor, of the high expectations made on a doctor, and of workload. On the basis of this insight, they developed and understanding of the circumstances under which doctors work even though the doctors are often stressed or despite their often unfriendly behaviour in the consultation, and they were obviously not afraid to address this observation.

SP 6: … “and, um, it happened (...), after an accident in stage that I had to go to the emergency room in the morning (...) and there I met a really ill-tempered surgeon, really out of sorts, and he was short with me and then I said: you probably had a tough night? And he says: do you really see that? I say: you are not happy and you are a bit grumpy with me, and then he started to apologize.” 

SP 1: …"but my perception and also my appreciation changed in relation to my doctor. So I am thinking: Oh, he really has time for me, and he engages in a detailed conversation in spite of the short time and small budget (…). 

##### Acting more self-confidently

Improved attentiveness and communication-related knowledge gained by SP training and activity seem to enable SPs to be more self-confident patients in the consultation. Many of the participating SPs felt encouraged to express their own needs to act more as “expert“ for their own matters. 

SP 11: “ I’m not afraid, afraid in quotation marks, if you wish, I am not reluctant to talk about stuff I notice, and I notice more than I did before”.

SP 9: “ I’ve become more ready to criticize, I dare to question more things, and well, this doesn’t always go so well, but – it has helped me because – the view I have of myself as a patient has changed, my self-confidence has increased”.

SP 9: …”and it has even happened that I take the liberty to say: I would like to say something as well”.

#### 3.3. Simulation and real life: three case studies

The three cases we selected for the second part of the analysis are examples of how the experience of being a SP and the feedback training may influence the everyday life of SPs, especially in their real life as patients. Lisa represents a group of SPs who acquired a new look at the health system and its actors and learned how to become more self-confident, more active and more communicative. Maria stands for SPs who had already been acquainted with the medical world and had a relaxed attitude towards doctors but keep a critical eye on medicine and doctors. Barbara is an outstanding case with a unique selling point insofar as she attributes her SP training a psychotherapeutic effect so that she meanwhile lost her fear of hospitals and doctors.

Lisa (SP6) : educator even outside the medical school

Lisa, 68 years old, has been working as an SP for four years and convincingly demonstrates self-confidence in the context of her work as SP as well as during the interview. This might be a result of her professional career as a teacher. Some years ago, she was diagnosed with a chronic disease and still has to visit several doctors frequently. However, in her everyday life she is only mildly affected by her disease. She is engaged in different social projects. Lisa is, among other things, active in the city’s cultural life and helps families with members who are suffering from dementia. It is her aim to transfer the skills she learned during the years working as a SP into her real life. 

Lisa felt a diffuse dissatisfaction with some doctors she met during the past years. Working as a SP enabled her for the first time to describe this feeling. Correspondingly, today she would give instant feedback in case of inappropriate behaviour and, if necessary, question a doctor’s procedures and explanations:

“…. um, and I’ve become highly critical of this meanwhile, (...) yes, I question everything.“

She exemplified this new ability by referring to a rather uncomfortable consultation with her GP, characterized by no greeting and a very short communication-style:

”…and then I just told her, well, you know, I’ve come to you here with a pounding heart, told you about what I feel, (...) I didn’t say that I’m an SP, that I didn’t say, of course, and I said, I actually expected that you’d be aware of me from the beginning. (...) And then she said goodbye with a handshake and said ’See you again soon’. That’s when I thought, maybe she’s learned something. (...) Well, I wouldn’t have had that courage before.“

Like other patients (see above), Lisa developed a better understanding of her GP after she learned more about a GP‘s everyday life and workload in our courses. The following quote shows how she changed her patient behaviour accordingly:

“…ok, when I have to visit a doctor now, I should be as precise as possible in describing what ails me, maybe write it up as a short list so I don’t waffle and also so I don’t overtax the doctor in his office“ „I as a patient need to have some empathy for the doctor, too.“

In her opinion, all patients should have her new knowledge about doctor’s night shifts, workload, and documentation requirements.

Maria(SP 10) a: simulation as a professional world of its own

Maria, 55 years old, formerly worked as the personal assistant of a head of department in the university hospital for many years. She has been working with us for three years. Apart from a little physical handicap she is an active person visiting the “university of the third age”. She is also working with the Samaritan emergency hotline for young people. 

As our interviews showed, many SPs generally described the doctor-patient-interaction as asymmetric. Maria, however, knows the routines of different medical environments very well and showed a sort of familiarity with medical institutions and personnel. This is more than the understanding of doctors other SPs reported in the course of their teaching activities. Instead, personality, professional skills, and teaching activities might all influence and motivate her more pragmatic view on, and a sober description of, doctors:

“At the time, or after the time that I um worked together with doctors, much had changed already. Umm, I think I can judge situations involving doctors more objectively now, because of this situation (working as secretary in a hospital). (...) A doctor is a doctor, nothing else, in my opinion umm, that is just the same as if there’d be a plumber in my flat.“

More than others, Maria realized the wide difference between teaching lessons and real life, perhaps due to her expertise and relaxed attitude towards doctors.

“Speaking as a patient, I just have to say that um I’ve experienced this at it’s worst and it makes me smile now, after the facts, particularly in the roles I’m in now; for instance, there was a situation where I had a doctor’s visit scheduled for myself and was an SP in the afternoon, and it was just exactly and glaringly the opposite”.

”….umm, I’d say the aim of this all would be the ideal case for the patient, I myself have witnessed this only very rarely, umm I just have to add this here, that was the reason for me (...) why I do this.“

The intimate knowledge from her professional career—and not empathy with doctors, as found in other SPs—seems to have been the reason she emphasized time as the most valuable and rare commodity in a GP’s real life:

”…umm, the teaching situation is set up so that the doctor is there for the patient and gives him the chance to express himself and find out what the problem is, which is of course also the goal of any normal office or any visit to a doctor’s, but very often the doctor needs to watch the time he’s taking, that is frequently very obvious“.

Barbara (SP3) : working as an SP as “therapy” 

Barbara, 59 years old, housewife and socially active within her community, has been working as an SP for three years. During the first part of the interview, she did not talk much about private matters and presented herself somewhat like a ‘blank slate’. Later in the interview, the analysis revealed that her motivation for becoming an SP was closely related to her painful experiences as a patient more than 30 years ago when she had to have a caesarean.

The gynecologist was very insensitive during a teaching-situation in which Barbara was involved (when medical students visited Barbara on the first day post-op). She remembered that the gynecologist shouted loudly, ‘now don’t make such a fuss!’ Barbara imitated the doctor’s intonation as closely as possible during the interview situation 35 years later; this illustrates how emotionally disturbing this incident was. These few words from the hospital doctor hurt her for years and prompted fear towards hospitals for a long time:

“When I’m telling you this now, my heart still begins to pound wildly and I just feel like blubbing again”. 

Since some of our courses took place in the same hospital in which Barbara had consulted the gynecologist 35 years before, she had to visit the house regularly as an SP. Obviously, both events, the regular visits to the hospital and the SP activity, helped her overcome her fears of the institution:

”Lost, this word lost, I’ve always felt so lost in hospitals. Um, and, um, was afraid that no one would help me there. ... Working as an SP has helped me accept this particular hospital that I visit twice a year when the exams are on (OSCE), I’ve always been terrified of hospitals. I can move in a hospital very casually now, that is something really exceptional for me, of course. This changed a lot (...), I’d guess I am about 70% less afraid of hospitals. That has changed.“

Moreover, because of her training and teaching activities, Barbara, similarly to other SPs, felt more self-confident in the medical context. Consequently, she now tries to support her own cause vis-à-vis the doctors.

 “If something happened to me like the situation I told you about. I’d never allow that to happen to me again.”

## 4. Discussion

Working as an SP enabled our interview partners to act more self-confidently in their real lives as patients, made them more sensitive towards a GP’s workload and duties, and may even introduce an intense change of the emotional state such as a reduction of anxiety. Feedback-training and learning new communication skills, similar to the training and work of our SPs, seem to be well-suited to empower patients in consultations with real doctors [18]. 

### 4.1. SP training as a way to empower patients?

Our results confirm findings from the United States and the Netherlands [10-12] where the experience of working as SPs was described in the context of becoming more attentive and critical and consequently feel more autonomous in medical environments. We know from two American studies [10], [13] that SPs acquire verbal skills that enable them to better explain their own perspective and needs in doctor-patient settings. Some of our interview partners also reported this learning process. As a result, they felt able to direct doctor-patient interactions more towards their own problems.

This raises the question of whether there is a “need to worry for GPs, because they face ‘supercritical’ patients”. In some instances, SPs apparently drew severe consequences, as reported by Woodward and Glivia-McConvey [13]: Some changed their GP after beginning working as SP, but the analysis of the interviews in our study did not show any similar consequences. On the contrary, our SPs—analogously to what was reported , had developed a better understanding of workload and pressure of time that physicians are exposed to, which surely is an effect of the better insight into the every-day work of GPs through the teaching situation. Moreover, as one SP suggested, it may also be a good idea to give feedback to doctors if they act insensitively. This kind of “education” of an insensitive GP was obviously successful in her case.

Our analysis did not show any negative effects on the well-being of the SPs. This is in contrast to the Dutch studies performed by Bokken [19] and colleagues, who detected exhaustion and/or physical complaints as a “side-effect” of simulation. The reason for these different results may be that our SPs had comparatively easy role plays and were trained and focused especially on history taking. They were neither physically examined nor required to perform complex clinical cases.

Training SPs and working as SPs may be a model of how to become an empowered patient. The results of our study highlight important components of this training and work, such as becoming more attentive and critical and feeling more autonomous in medical environments [20]. In addition, our SPs acquired verbal skills that helped them to better explain their own needs in clinical settings, and they learned to give feedback.

#### 4.2. SP and real-life patient

In spite of identical training, the teaching activities of Barbara, Lisa, and Maria led to different consequences in their daily lives:

1. *Barbara’s *case (working as an SP as a psychosocial therapy) shows a constellation that to our knowledge has not been described to date: a traumatic event that causes fear of and aversion to hospitals was changed by her time as an SP. Barbara transformed this negative event into a motivation to be part of a teaching team in a medical school, obviously a successful coping strategy to overcome her fears. It is important to emphasize that it was not this negative event that motivated Barbara to become an SP, but the job helped her in the real life as a patient, and this made her SP activity more important to her.
2. *Lisa* represents another type of SP: educator even outside the medical school. As a highly skillful patient, she had the motivation to transport her expertise into her everyday-life (outside the medical school). The quality standards she learned during teaching lessons should be also standard in her real relationship with physicians. This type of SP sets out to change the students’ as well as a doctor’s behaviour, not only for short duration, as described by Rubin [9], but lastingly. Lisa’s behaviour also corresponds to the proactive engagement with clinicians as a strategy for empowerment in the “patient-as-professional” model [21]. For example, one patient in an Australian study, which explored this model, described how she always had to keep her GP informed all the time and questioning about her medication and, in the end, to manage the health care team [22]. Other SPs in our study seemed to act similarly in the world outside the medical school. It is only a marginal remark in the context of our study that even a person who feels self-confident in the life-world, like Lisa does, obviously needs the training and experience of an SP to become self-confident in the world of medicine.
3. *Maria’s* motivation to work as an SP (simulation as a professional world of its own) lacks an intimate touch. It seems first and foremost based on a pedagogic ethos, probably originating in her medical background. Consequently, she reported only few personal events and distinguished between the teaching situation and being a patient in real life. She did not feel influenced in her own role as patient by her teaching knowledge and vice versa. But she emphasized more strongly than anyone else the need of teaching communicative skills. Maria’s interview was not so much a narrative but an argumentative and rational presentation, more from the perspective of an expert than from that of a patient. She reported in unemotional language that some GPs in her experience lack communication skills or a GP’s time constraints are barriers for a strong patient-clinician relationship. This can be interpreted as Maria’s way to demonstrate herself as a sort of ‘professional SP’ or ‘patient-as-professional’ [21] including the ‘duty’ to share one’s knowledge with others [13], [14], [23], which emphasizes the social context of empowerment.

#### 4.3. Strengths and weaknesses of the study

The interviews used open-ended questions, which afforded our interview partners the opportunity to set their own focus on facts and experiences that were relevant in their work as SP and in their real life. Data analysis with a multi-disciplinary team provides a good basis for detecting and examining relevant events and experiences.

While we certainly have a complete picture of our own SP staff, the results are not representative for SPs in Germany. Training and teaching in other medical schools may be different and, thus, the knowledge of SPs in other settings and the consequences for their life may likewise be different. The sampling of our interview partners and analysis did not follow the concept of theoretical sampling, so that we cannot be certain to have reached data saturation [24]. Moreover, our interview partners were self-selected and may have been inclined to report in a positive manner.

## 5. Conclusions and implications for practice

Personal background, experiences, and motivation of SPs were mixed to various degrees, but were predictably different for each SP. Role-playing may support acting more self-confidently or improve the understanding of doctors, or it may motivate SPs to share knowledge, insights, and ideas with others, as in the case of Maria. 

As we know from other studies [17], fear, ignorance, and reluctance to ask the doctor are general emotions patients experience in their medical consultations. Instead of appealing to doctors to act more patient-oriented, the training in our course may be a model not for SPs alone, especially the part of becoming familiar with the rules of good (and poor) communication and giving professional feedback. The changes described in this as well as in other studies could also be read as one way to enact the ‘patient-as-professional’ concept [22] with the aim to empower patients and guide their relation and communication with healthcare professionals [19]. 

If the empowerment of SP through their job also is true at other locations, this knowledge should be used in casting and teaching situations. SPs and trainer should know about these findings and then would be able to include them during SP-training.

Working as an SP seems to empower patients. Future research should explore how some of the SP teaching components can be transferred into other educational settings to support and train real patients who are interested to take a more active role in health care consultations. 

## Acknowledgements

We thank the SPs we interviewed for their attendance. We thank Kathrin Püschel for helping with the data analysis.

## Authors

PD Dr. ANNE SIMMENROTH-NAYDA, is working as a researcher and family physician. Areas of interest: medical education, simulated patients, clinical medicine.

GABRIELLA MARX, PhD, is working as a researcher in the Palliative Medicine of Göttingen Medical Center. 

THORSTEN LORKOWSKI, MD, wrote his doctoral thesis about simulated patients in medical teaching. He is working as resident in the Buxtehude Hospital.

WOLFGANG HIMMEL, PhD, works as senior lecturer in health sciences. Areas of interest: doctor-patient relationship; health services research.

## Competing interests

The authors declare that they have no competing interests.
